# Enhancing Patient Management and Financial Efficiency: Adherence to National Institute for Health and Care Excellence (NICE) Guidelines for Pediatric Distal Radius Buckle Fractures

**DOI:** 10.7759/cureus.44198

**Published:** 2023-08-27

**Authors:** Jehan Zaib, Jawad Ahmad, Krishna Kumar

**Affiliations:** 1 Trauma and Orthopedic, Dudley Group of National Health Service (NHS) Hospitals, Birmingham, GBR; 2 Trauma and Orthopaedics, Hull University Teaching Hospitals, Hull, GBR; 3 Trauma and Orthopaedics, Cumberland Infirmary Carlisle, Carlisle, GBR

**Keywords:** wrist fractures, nice guidelines, paediatric distal radius fractures, buckle fractures, torus fractures

## Abstract

Introduction: Pediatric distal radius buckle fractures are commonly encountered in the emergency department (ED) and are considered non-complex and stable injuries. The National Institute for Health and Care Excellence (NICE) guidelines recommend managing these fractures with a soft cast and discharging patients directly from the ED. However, prevailing practices often involve rigid casts and follow-up clinic visits, leading to unnecessary congestion, prolonged waiting times, excessive radiographic examinations, and frequent cast changes, resulting in additional financial burdens on hospitals.

Methods and Materials: We conducted an initial audit over a 6-month period at Hull University and Teaching Hospitals, reviewing 184 pediatric distal radius fractures, of which 84 were buckle fractures in children under 12 years old. Data on demographics, subsequent clinic visits, treating doctor's grade, additional radiographs, initial and final treatment approaches, and cast change frequency were collected. After the initial audit, NICE guideline compliance was promoted through the education of parents and healthcare providers. A second audit was performed on patients within the following 6-month period.

Results: This study assessed the management of pediatric distal radius buckle fractures in a cohort of 84 patients. 39/84 (46.4%) of patients sought medical attention within one week of sustaining the injury, with 33/84 individuals being discharged during their first visit, either by consultants or registrars. Most patients (69/84) required only a single X-ray examination in the ED, while some needed two or three X-rays during their evaluation. However, after implementing NICE guidelines, in the second audit cycle, 62 out of 64 were discharged directly from the ED, with 42 receiving focal rigidity casts (FRCs) removed at home and 10 discharged with simple crepe bandages.

Conclusions: This closed-loop audit effectively showcased that adherence to NICE guidelines yielded better patient management by avoiding unnecessary visits, radiographs, and platers. The adoption of the guidelines leads to the conservation of time and resources.

## Introduction

Pediatric distal radius buckle fractures are considered non-complex and inherently stable fractures, comprising a significant proportion of fractures encountered in the emergency department (ED) setting [1]. Despite clear recommendations provided by the National Institute for Health and Care Excellence (NICE) guidelines, which suggest managing these fractures with a soft cast and discharging patients directly from the ED [2], there is significant variability in practice concerning the necessity of wrist immobilization and clinical follow-up for children with these fractures, both nationally and internationally. However, torus fractures heal rapidly, and there is growing consideration that simple, removable splints could be safe and effective alternatives to casts [3]. A recent trial has demonstrated that even simple bandages are sufficient for treating these types of injuries [4].

 The variable practice, not guided by guidelines, contributes to unnecessary congestion in pediatric clinics, prolonged waiting times, unwarranted radiographic examinations, and, at times, frequent cast changes. Consequently, these factors impose an additional financial burden on hospitals [5].

We audited our management of pediatric distal radius buckle fractures over a period of 6 months against NICE guidelines and then closed the loop by re-auditing it.

## Materials and methods

Between February and July 2019, we conducted our initial audit at Hull University and Teaching Hospitals. The ethical approval was taken before conducting both audits by the hospital audit team. The identifiers of all the patients were kept anonymized. Retrospective data collection was performed by reviewing electronic records specifically labeled as distal radius fractures. A total of 184 pediatric distal radius fractures were reviewed, with 85 cases identified as buckle fractures in children under the age of 12. Children less than 12 years old with classical buckle fracture in distal 1/3 of the radius were included. Children having angulated, greenstick, and ipsilateral injuries were excluded. Demographic information, laterality, subsequent visits to the fracture clinic, the discharging doctor's grade, the number of additional radiographs, initial and final treatment approaches, as well as the frequency of cast changes, were recorded and analyzed.

In August 2019, data were presented, and information was disseminated to the department and the emergency department (ED) with the aim of aligning our practices with the NICE guidelines. This involved educating parents about the fracture type, providing instructions on cast removal, and offering them the option to forgo further clinic visits. The safety brochures have been in place since 2017 but were not being used effectively. It was made sure through email reminders that everyone in the fracture clinics was aware of the NICE guidelines for the treatment of buckle fractures.

A second audit was performed on patients between September 2019 and February 2020 and the same variable was recorded as before. The financial implication of extra visits, radiographs, and casts was calculated after gaining an estimated amount from the procurement department.

## Results

**Figure 1:**
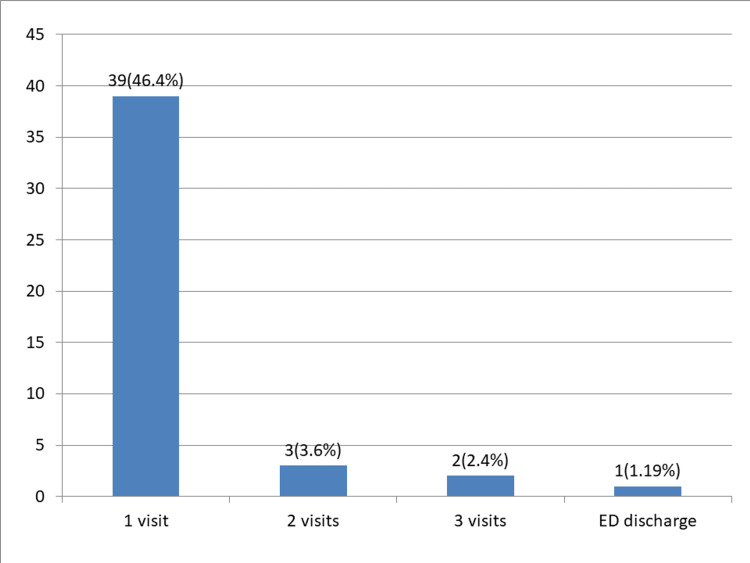
Visit frequency in fracture clinic: first audit.

**Figure 2:**
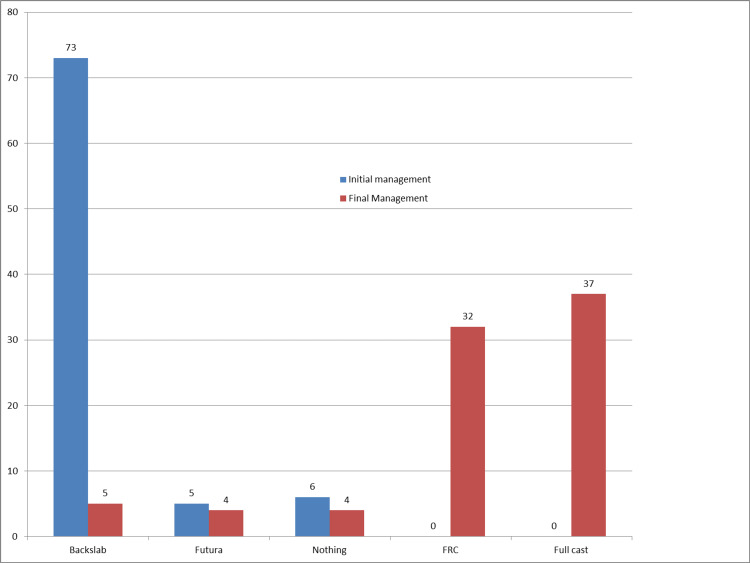
Treatment at the time of initial and final visit: first audit.

The basic demographics for the 84 patients are presented in Table 1. Among them, 81 patients sought medical attention within 1 week of sustaining the injury, while four patients presented after 1 week. The number of patient visits is shown in Figure 1. Notably, 69 patients underwent a single X-ray examination in the emergency department (ED) without the need for subsequent repeat X-rays. However, 12 patients underwent two X-rays, and an additional four patients required three X-rays. The initial and final management approaches for patients are shown in Figure 2. Out of the total patient population, 33 individuals were discharged during their first visit, with 18 being discharged by consultants and 15 by registrars.

**Table 1:** Basic demographics from both audits.

Age (mean)	7.76 (±2.39) Range (1-11)
Gender	
Male	56(37.8%)
Female	92(62.2%)
Hand dominance	
Right	45(30.4%)
Left	103(69.6%)

**Figure 3:**
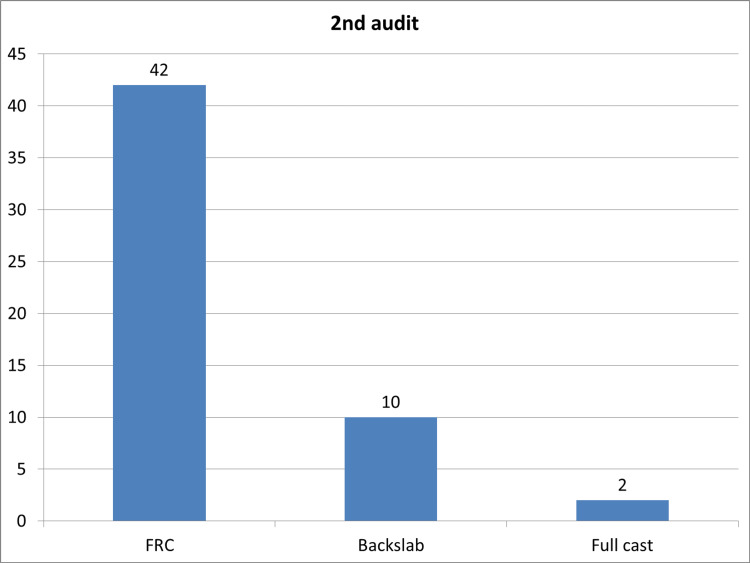
Initial management during the period of second audit.

In the second cycle, a total of 64 patients were evaluated in the ED. Out of these patients, 62 were discharged directly from the ED. Among them, 42 were provided with a focal rigidity cast (FRC) which was removed at home, 10 were given backslabs, and 10 were discharged with a simple crepe bandage. The type of treatments administered during the second cycle is shown in Figure 3. Additionally, two patients returned to the plaster room, and one patient returned to the fracture clinic and was discharged during the same visit.

## Discussion

Implementing NICE guidance offers numerous benefits for healthcare stakeholders. Patients, carers, and service users receive evidence-based care, promoting accountability and improved health outcomes. Healthcare professionals gain confidence in decision-making while efficiently allocating resources for better health outcomes. Organizations benefit from reduced claims and financial costs, aligning service provision with national priorities, thus promoting community well-being and prosperity. The implementation of national guidelines can encounter obstacles at multiple levels, spanning from the national, including regulatory factors, to the organizational level, characterized by insufficient staff, resources, and managerial direction, as well as challenges at the individual level [6]. In our study, the predominant barriers to implementation were at the individual level, primarily stemming from a lack of knowledge and inadequate managerial direction.

The effective adoption of NICE guidelines within our research has resulted in a noteworthy decrease in the frequency of visits, unnecessary radiographs, and unneeded treatments at fracture clinics. These clinics frequently grapple with issues of overbooking and prolonged waiting periods. This outcome is corroborated by a separate study [7], wherein a reduction in clinic visits was observed. However, our study has demonstrated a more substantial reduction in visits compared to this prior research. Specifically, in the mentioned study, the visits decreased from 59 to 39 visits, whereas our study has shown a remarkable outcome with only one patient requiring a return visit to the fracture clinic, and that too for reassurance purposes.

This positive outcome after the implication of NICE guidelines resulted in a notable financial impact, saving a substantial amount over a 6-month period by avoiding unnecessary visits, radiographs, and plaster changes. In the first audit, the extra visits, radiographs, and plaster changes resulted in a cost of 18,078£ just over a period of 6 months. The detailed breakup of the finances is mentioned in Table 2. A study conducted in Leicester [8] has indicated that the financial implications of managing these fractures within a hospital setting are more substantial when compared to home-based management. Nevertheless, it is worth noting that this particular study did not delve into the detailed analysis of financial expenses as comprehensively as our own research has.

**Table 2:** Financial impact when NICE guidelines were not followed. NICE, National Institute for Health and Care Excellence

Extra visits to clinics	84 x 132	£11088
Extra visits after first week	45 x 132	£5940
Change of casts	42 x 25	£1050
Total		£18078

The benefits of reducing unnecessary visits extend beyond financial savings. Patients and parents are relieved of the additional burden of scheduling and attending extra appointments, which can be particularly challenging for school-going children [9].

The compliance of medical staff and the reassurance provided to patients played pivotal roles in achieving these positive results. Although the guidelines and safety brochures were already in place, the study highlighted the importance of proper adherence by medical personnel to ensure successful outcomes.

The primary strength of this study lies in its successful demonstration of how the implementation of straightforward guidelines can objectively reduce the financial burden and contribute to the standardization of management practices. However, several limitations warrant consideration. The retrospective nature of the study and its confined timeframe of six months restrict the ability to capture potential seasonal variations comprehensively. Furthermore, the study's emphasis was primarily on clinic visits, fracture management methods, and financial outcomes, neglecting the systematic collection of patient-reported outcomes like pain and satisfaction. This gap limits a comprehensive understanding of the patient's perspective.

## Conclusions

The study's findings underscore the significance of implementing and adhering to simple but well-thought NICE guidelines, as they offer substantial benefits for both patients and healthcare providers. Overall, compliance with NICE guidelines resulted in more efficient and cost-effective fracture management.
